# Comparative effects of intraduodenal amino acid infusions on food intake and gut hormone release in healthy males

**DOI:** 10.14814/phy2.13492

**Published:** 2017-11-15

**Authors:** Robert E. Steinert, Sina S. Ullrich, Nori Geary, Lori Asarian, Marco Bueter, Michael Horowitz, Christine Feinle‐Bisset

**Affiliations:** ^1^ Adelaide Medical School University of Adelaide Adelaide South Australia Australia; ^2^ NHMRC Centre of Research Excellence in Translating Nutritional Science to Good Health University of Adelaide Adelaide South Australia Australia; ^3^ Department of Surgery Division of Visceral and Transplantation Surgery University Hospital Zürich Zürich Switzerland; ^4^ Department of Psychiatry Weill Medical College of Cornell University New York New York; ^5^ Department of Medicine‐Immunobiology Robert Larner College of Medicine University of Vermont Burlington Vermont

**Keywords:** Cholecystokinin, energy intake, glutamine, humans, leucine, phenylalanine, tryptophan

## Abstract

In contrast to the many studies of the effects of individual amino acids (AAs) on eating, no studies have compared the effects of different AAs on eating and underlying preabsorptive gastrointestinal mechanisms. To compare the effects of intraduodenal infusions of l‐tryptophan (TRP), l‐leucine (LEU), l‐phenylalanine (PHE) and l‐glutamine (GLN) on appetite, gastrointestinal hormone responses (including ghrelin, cholecystokinin (CCK), peptide YY (PYY) and glucagon‐like peptide‐1 [GLP‐1]), glycemia (glucagon, insulin and glucose) and test meal size in healthy males, we retrospectively analyzed data from four published independent, randomized, double‐blind, placebo‐controlled studies of 90‐min intraduodenal infusions of the individual AAs. The designs of the studies were identical, except the dose of TRP (0.15 kcal/min) was lower than that of the other AAs (0.45 kcal/min) because higher doses of this AA were not well tolerated. TRP and LEU decreased intake more than PHE (reductions relative to control, ~219 ± 68, ~170 ± 48 and ~12 ± 57 kcal, respectively), and TRP decreased intake more than GLN (~31 ± 82 kcal). These effects of TRP and LEU versus GLN, but not versus PHE, were paralleled by greater decreases in plasma ghrelin, and increases in CCK, concentrations. TRP increased PYY more than GLN or LEU, but not PHE. LEU increased PYY less than PHE. No significant differences were detected for GLP‐1. PHE increased glucagon more than TRP or LEU, and increased insulin more than TRP. Under our experimental conditions, intraduodenal TRP and LEU were more satiating than PHE and GLN. The greater satiating efficacy of LEU versus PHE was significantly dissociated from the effects of these AAs on PYY, while the greater satiating potency of TRP versus PHE was significantly dissociated from the effects of these AAs on insulin and glucagon. In contrast, ghrelin and CCK, and potentially other mechanisms, including central sensing of individual AAs, appear to be stronger candidate mechanisms for the relative satiating effects obtained.

## Introduction

There is evidence that protein inhibits eating and appetite more than carbohydrate or fat in healthy‐weight and obese individuals (Batterham et al. 2003; Brennan et al. 2012), a notion reflected also in the protein leverage hypothesis (Simpson and Raubenheimer 2005; Gietzen and Aja 2012). The eating‐inhibitory effects occur, dependent on energy balance, both after acute administration, for example, within single meals (Rolls et al. 1988; Batterham et al. 2006; Brennan et al. 2012), and in individuals adapted to high‐protein diets (Due et al. 2004; Weigle et al. 2005; Westerterp‐Plantenga et al. 2009; Martens and Westerterp‐Plantenga 2014). In addition, protein is increasingly recognized for its beneficial effect on postprandial glycemic control in type 2 diabetes (Ma et al. 2009) and its capacity to preserve muscle mass during weight loss (Pasiakos et al. 2013). Accordingly, there is increasing interest in the use of protein supplements and high‐protein diets in the management of obesity with and without type 2 diabetes (Larsen et al. 2010).

A variety of mechanisms may contribute to the inhibitory effects of protein on eating. Postabsorptive effects include the thermic effect of protein (Westerterp‐Plantenga et al. 1999), increased intestinal gluconeogenesis and associated glucose sensing in the portal vein (Mithieux et al. 2005), and central sensing of absorbed amino acids (AAs), a possibility originally proposed by Mellinkoff as the aminostatic control of eating (Mellinkoff et al. 1956). Preabsorptive effects may also contribute to the satiating efficacy of protein. These include changes in gastrointestinal (GI) motor function, in particular the rate of gastric emptying, which affect the degree of gastric distension, and secretion of GI hormones, such as ghrelin, cholecystokinin (CCK), glucagon‐like‐peptide (GLP‐1), and peptide tyrosine tyrosine (PYY), all of which are thought to contribute to the control of eating (Steinert et al. 2017).

The GI responses to proteins appear to be affected by their AA compositions, although which protein type is most satiating remains unclear (Hall et al. 2003; Anderson et al. 2004; Diepvens et al. 2008). For example, Hall et al. (2003) reported in healthy‐weight subjects that a preload of whey protein, which is rich in branched‐chain AAs, decreased eating, increased plasma levels of CCK and GLP‐1, and slowed the initial rate of gastric emptying substantially more than an isoenergetic preload of casein. Similarly, administration of individual AAs appears to lead to differential eating and GI responses. Oral loads and intraduodenal (ID) infusions of l‐tryptophan (TRP), l‐phenylalanine (PHE) or l‐leucine (LEU) reduced eating and increased plasma CCK in several studies (Ballinger and Clark 1994; Carney et al. 1994; Edelbroek et al. 1994; Steinert et al. 2014, 2015a), whereas no effects on eating were detected in studies of oral or intragastric preloads of l‐proline (Nuttall et al. 2004), l‐isoleucine (Nuttall et al. 2008; Ullrich et al. 2016) or l‐lysine (Kalogeropoulou et al. 2009). In addition, we (Chang et al. 2013; Steinert et al. 2014, 2015a,b) and others (Greenfield et al. 2009) reported apparently differential effects of oral loads and ID infusions of AAs, including l‐glutamine (GLN), on plasma levels of GLP‐1 and antropyloroduodenal motility. In contrast to the several studies of the effects of different proteins, however, none of the latter studies compared the magnitude of effects of the different AAs. Therefore, to better understand the relative effects of individual AAs on eating, and to gain insights into the potential mechanisms that may lead to differential satiating effects, we compared the effects of premeal ID infusion of TRP, LEU, PHE or GLN on appetite, GI‐hormone responses, and meal size (in kcal [1 kcal = 4.184 kJ]), using data from identically‐designed, randomized, double‐blind, placebo‐controlled tests that have in part been previously published (Steinert et al. 2014, 2015a,b).

## Materials and Methods

### Subjects

Four groups of healthy, normal‐weight men (mean ± SD; TRP study: *n* = 10, age (years): 26.6 ± 8.6 (range 18–42), BMI (kg/m^2^): 22.5 ± 2.1 (range 18.7–24.7); LEU study: *n* = 11, age: 24.9 ± 6.9 (range 18–44), BMI: 21.9 ± 1.6 (range 18.9–23.9); PHE study: *n* = 10, age: 23.6 ± 6.2 (range 18–40), BMI: 22.6 ± 2.1 (range 19.4–25.3); GLN study: *n* = 9, age: 30.3 ± 13.2 (range 19–48), BMI: 22.5 ± 2.3 (range 18.5–25.3) were studied (Fig. S1). In each study, the number of subjects was determined by power calculations based on our previous studies of the effects of ID glucose or fatty acids on food intake and GI motor and hormone responses (Feltrin et al. 2004; Pilichiewicz et al. 2007). Exclusion criteria were smoking, consumption of >20 g of alcohol/day, any medical condition, chronic GI symptoms, and the use of medications known to affect GI function or appetite. Once included, participants were assigned to a treatment order, which was generated by an online tool for balanced randomization (www.randomization.com) and performed by a research officer who was not involved in data analysis. The study protocols were approved by the Royal Adelaide Hospital Research Ethics Committee and carried out in accordance with the Declaration of Helsinki. Each subject provided informed, written consent prior to their enrolment.

### Study design and protocol

In each of the four studies, subjects were studied on three occasions, each separated by 3‐10 days, on which they received, in randomized, double‐blind fashion, 90‐min ID infusions of LEU, PHE, GLN (total loads of 3.3 or 9.9 g, i.e., 0.15 or 0.45 kcal/min, or isovolemic control infusions), or TRP (loads of 1.7 or 3.3 g, i.e., 0.075 or 0.15 kcal/min, or control). Only data collected in response to the higher dose for each AA were included in the current analysis. The LEU, PHE, and GLN loads were based on a report that 10 g of PHE was well tolerated and inhibited eating and stimulated plasma CCK in healthy subjects (Ballinger and Clark 1994). The TRP dose was based on a previous study in which 3 g of TRP decreased hunger and slowed gastric emptying (Carney et al. 1994), and on our own preliminary studies (Steinert et al. 2014) in which ID infusion of 0.1 kcal/min TRP was well tolerated, but infusions above 0.2 kcal/min led to reports of nausea or dizziness. AA solutions were prepared by dissolving crystalline AA (PureBulk, Roseburg, OR), 118.3 mg CaCl_2 _× 2H_2_O and NaCl, to achieve isotonic [300 mOsm] solutions in 405 mL distilled water. The isotonic control solution contained 118.3 mg CaCl_2 _× 2H_2_O and 4.6 g NaCl in 405 mL distilled water for TRP, PHE, and GLN and, 124.8 mg CaCl_2 _× 2H_2_O and 3.2 g NaCl in 428 mL distilled water for LEU, due to the lower solubility of the latter. Solutions were administered at a rate of 4.5 (TRP, PHE, GLN) or 4.75 (LEU) mL/min. Solutions were prepared by a research officer who was not involved in the performance of the study or analysis of the data, and were covered to blind the study subject and the investigators performing the studies.

Subjects were instructed to abstain from alcohol and strenuous exercise for 24 h and were provided with a standardized meal (Beef Lasagna, McCain Food, Wendouree, Victoria, Australia, total energy content: 1160 kcal) to be consumed at 19:00 h on the evening before each visit. Subjects then ate or drank nothing, except water, until they arrived at the laboratory at 08:30 h the following day. On arrival, each subject was intubated with a manometric catheter (total length: 100 cm; Dentsleeve International, Mui Scientific, Mississauga, Ontario, Canada) used for monitoring antropyloroduodenal pressures and ID administration of the solutions (Steinert et al. 2014, 2015a,b). The latter were infused through a side‐hole located ~14.5 cm distal to the pylorus. An intravenous cannula was placed into a right forearm vein for blood sampling. Once the catheter was positioned correctly (Steinert et al. 2014, 2015a,b), during phase I of the interdigestive migrating motor complex, baseline blood samples at *t *= −10 and 0 min were taken, and ID AA or control infusions were given from 0 to 90 min. Blood samples were collected every 15 min from 0 to 90 min, and perceptions of appetite and GI symptoms were evaluated at the same time points (Parker et al. 2004). At *t *= 90 min, the infusion was terminated and the catheter removed. Subjects were then offered a standardized, cold, buffet‐style test‐meal, the composition of which has been described in detail previously (Nair et al. 2009), and invited to consume as much food as they wished until they felt comfortably full, for a maximum of 30 min (*t* = 90 to 120 min). After completion of the meal, at *t* = 120 min, a final blood sample was collected and a VAS questionnaire administered. Subjects were then free to leave the laboratory.

### Measurements

#### Food intake and appetite and GI perceptions

Energy intake (kcal) during the meal was calculated from the amount of food eaten (g), measured by weighing each food item before presentation and at the end of the meal, using commercial software (FoodWorks 7.0; Xyris Software, Highgate Hill, Queensland, Australia). Perceptions of fullness, hunger, prospective food consumption and desire to eat, as well as nausea and bloating, were quantified using validated 100‐mm visual analog scales (VAS) questionnaires (Parker et al. 2004).

#### Blood glucose and plasma hormone concentrations

Blood samples were collected into ice‐chilled EDTA tubes. Blood glucose was determined using a portable glucometer (Medisense Precision QLD; Abbott Laboratories, Bedford, MA). Plasma was separated by centrifugation at ~1830 ***g*** for 15 min at 4°C within 15 min of collection and stored at −70°C until assayed. Plasma total ghrelin (pg/mL) was measured by radioimmunoassay (Phoenix Pharmaceuticals, Mountain View, CA). Intra‐ and inter‐assay coefficients of variation (CVs) were ~5% and 15%, respectively. The detection limit was 44 pg/mL. Plasma CCK (pmol/L) was measured by radioimmunoassay after ethanol extraction using an adaptation of the method of Santangelo et al. (1998). Intra‐ and inter‐assay CVs were ~8% and 13%, respectively. The detection limit was 1 pmol/L. Plasma total GLP‐1 (pmol/L) was measured by radioimmunoassay (Millipore, Billerica, MA). Intra‐ and inter‐assay CVs were ~5% and 7%, respectively. The detection limit was 3 pmol/L. Plasma total PYY (pg/mL) was measured by radioimmunoassay (Linco Research, St. Charles, MO). Intra‐ and inter‐assay CVs were ~5% and 7%, respectively. The detection limit was 10 pg/mL. Plasma insulin (mU/L) was measured by an ELISA assay (Mercodia, Uppsala, Sweden). Intra‐ and inter‐assay CVs were ~3% and 9%, respectively. The detection limit was 1 mU/L. Plasma glucagon (pg/mL) was measured by radioimmunoassay (Millipore). Intra‐ and inter‐assay CVs were ~3% and 6%, respectively, and the detection limit was 20 pg/mL.

### Data and statistical analysis

Baseline (“0”) values were calculated as means of values obtained between *t* = −10 and 0 min. Incremental areas under the curve (iAUCs) were calculated for each AA and its control infusion under the profiles (*t* = 0 to 90 min) of VAS scores, blood glucose and plasma hormone concentrations. For all parameters (except food intake), data were then expressed as change in iAUC for each AA relative to control infusion. These delta iAUCs are presented in the Results section.

Data were analyzed with SPSS software (version 19.0, SPSS, Chicago, IL) using planned comparisons, which better focuses statistical power on specific comparisons of interest than does factorial ANOVA. Two‐way analyses of variance, with delta iAUCs as the first factor and AA type as the second factor, were performed to generate experiment‐wide residual errors, which were used to compute standard errors of the difference (SED) and *t*‐tests, whose significances were determined using the Bonferroni–Hochberg method (Hochberg 1988), with minimum experiment‐wide two‐tailed alpha levels of 0.05. Experiment‐wide SEDs were 64 kcal for meal size, 318 mm × min for prospective food consumption, 1594 pg/mL × min for ghrelin, 14 pmol/L × min for CCK, 222 pmol/L × min for GLP‐1, 716 pg/mL × min for PYY, 9.5 mmol/L × min for blood glucose, 24 mU/L × min for insulin and 318 pg/mL × min for glucagon. Five comparisons were performed to test whether GLN or PHE inhibit eating differently than TRP or LEU, which each inhibited eating when tested individually (Steinert et al. 2014, 2015a), and whether TRP inhibits eating differently than LEU: (1) GLN versus TRP, (2) GLN versus LEU, (3) PHE versus TRP, (4) PHE versus LEU, and (5) TRP versus LEU. The GLN versus PHE comparison was not done because neither of these AA inhibited eating when tested individually. The same five comparisons were done for all other measurements. Data are presented as means ± SEM.

## Results

### Appetite and GI perceptions

**Figure 1:**
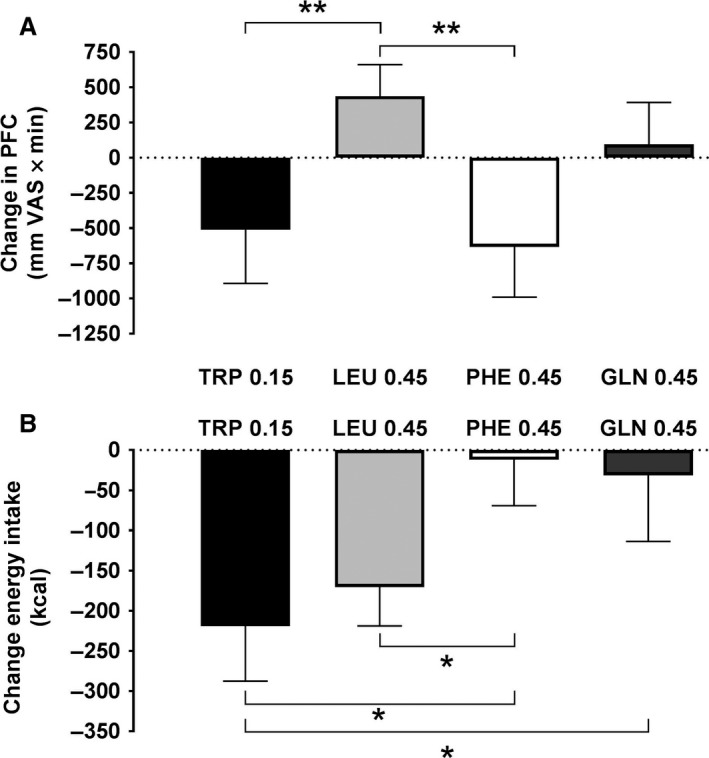
Changes in (A) food intake at a buffet meal and (B) premeal visual analog scale (VAS) ratings of prospective food consumption (PFC) in response to 90‐min intraduodenal infusions of l‐tryptophan (TRP) at 0.15 kcal/min, or l‐leucine (LEU), l‐phenylalanine (PHE) or l‐glutamine (GLN) at 0.45 kcal/min. Energy intake data are changes from respective control infusions, means ± SEM; PFC data are 90‐min incremental areas under the curve relative to control (delta iAUCs); *n* = 10 for TRP and PHE, *n* = 9 for GLN, and *n* = 11 for LEU. Data were analyzed using a planned comparison approach. Two‐way analyses of variance, with delta iAUCs as the first factor and AA type as the second factor, were performed to generate experiment‐wide residual errors, which were used to compute standard errors of the difference (SED) and t‐tests, whose significances were determined using the Bonferroni–Hochberg method (Hochberg 1988), with minimum experiment‐wide two‐tailed alpha levels of 0.05. Five comparisons were performed to test whether GLN and PHE inhibit eating similarly to TRP and LEU and whether TRP inhibits eating similarly to LEU: (1) TRP versus LEU, (2) TRP versus GLN, (3) TRP versus PHE, (4) LEU versus GLN, and (5) LEU versus PHE. *P < 0.05, **P < 0.01

TRP (−512 ± 381 mm × min, relative to control infusion) and PHE (−634 ± 356) each decreased delta iAUC for prospective food consumption significantly more than LEU (436 ± 223; *P* < 0.05 and *P *< 0.01, respectively); the difference between LEU and GLN (94 ± 298) was not significant (Fig. 1A). The planned comparisons failed to reveal significant differences in the delta iAUCs for fullness, hunger, desire to eat, nausea or bloating during TRP, LEU, PHE or GLN infusion (data not shown).

### Energy intake

Both TRP (219 ± 68 kcal) and LEU (170 ± 48 kcal) decreased energy intake more than PHE (12 ± 57 kcal; *P* < 0.05), and TRP decreased energy intake more than GLN (31 ± 82 kcal, *P* < 0.05) (Fig. 1B). The mean reduction in energy intake appeared greater with LEU than with GLN, but the difference was not significant.

### Plasma ghrelin, CCK, GLP‐1 and PYY

Individual time profiles for plasma ghrelin, CCK, GLP‐1, PYY, insulin, glucagon and blood glucose are shown in Figures [Link], [Link], [Link], [Link], [Link], [Link], [Link]. The results of the planned comparisons of delta iAUCs are reported below.

#### Ghrelin

**Figure 2:**
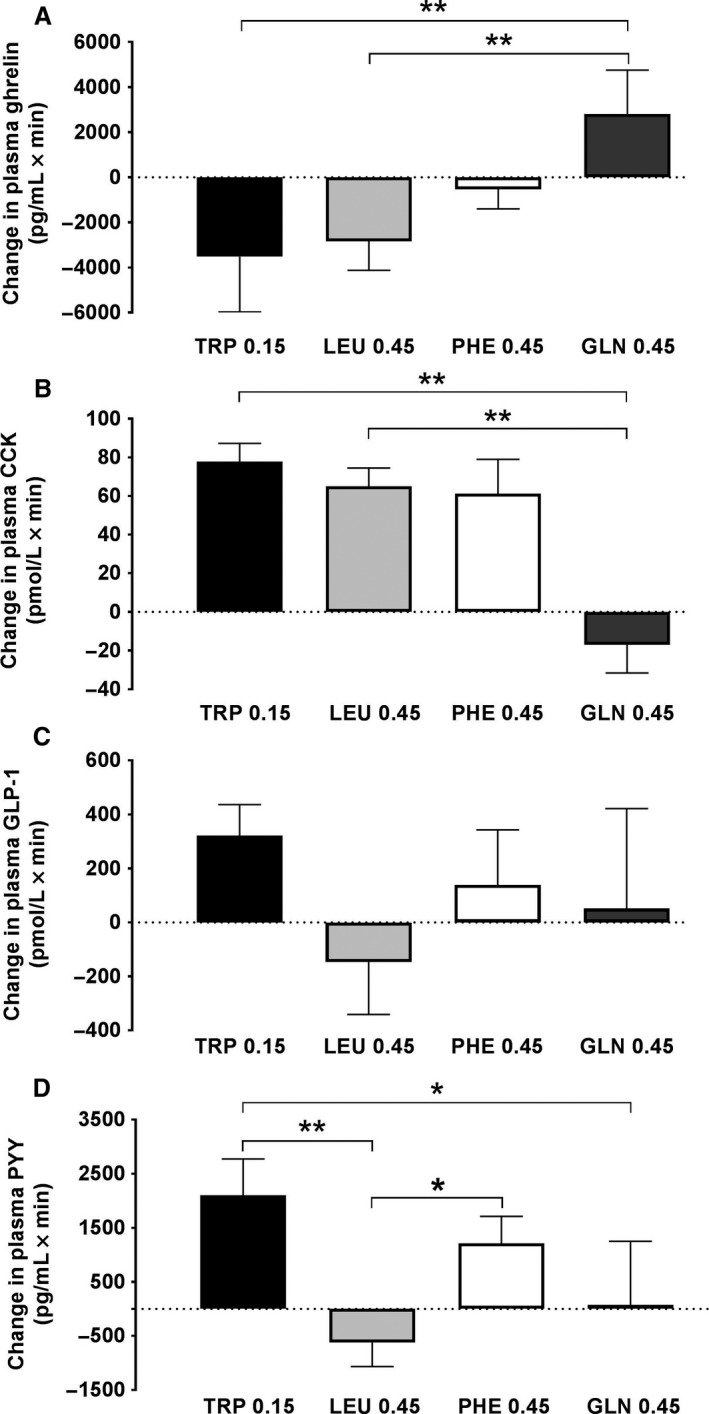
Changes in plasma ghrelin (A), cholecystokinin (CCK) (B), glucagon‐like peptide‐1 (GLP‐1) (C) and peptide YY (PYY) (D) during 90‐min intraduodenal infusions of l‐tryptophan (TRP) at 0.15 kcal/min, or l‐leucine (LEU), l‐phenylalanine (PHE) or l‐glutamine (GLN) at 0.45 kcal/min. Data are changes from respective control infusions, expressed as 90‐min incremental areas under the curve (delta iAUCs), means ± SEM; *n* = 10 for TRP and PHE, *n* = 9 for GLN, and *n* = 11 for LEU. Data were analyzed using a planned comparison approach. Two‐way analyses of variance, with delta iAUCs as the first factor and AA type as the second factor, were performed to generate experiment‐wide residual errors, which were used to compute standard errors of the difference (SED) and *t*‐tests, whose significances were determined using the Bonferroni–Hochberg method (Hochberg 1988), with minimum experiment‐wide two‐tailed alpha levels of 0.05. Five comparisons were performed to test whether GLN and PHE inhibit eating similarly to TRP and LEU and whether TRP inhibits eating similarly to LEU: (1) TRP versus LEU, (2) TRP versus GLN, (3) TRP versus PHE, (4) LEU versus GLN, and (5) LEU versus PHE. *P < 0.05, **P < 0.01

Both TRP (−3524 ± 2453 pg/mL × min) and LEU (−2842 ± 1282) reduced plasma ghrelin delta iAUC significantly more than GLN (2804 ± 1954; *P* < 0.01 for both), but the differences between TRP and PHE (−534 ± 862) and between LEU and PHE were not significant (Fig. 2A).

#### CCK

Both TRP (77 ± 9 pmol/L × min) and LEU (65 ± 9) increased plasma CCK delta iAUCs more than GLN (−17 ± 15; *P* < 0.01 for both), but the differences between TRP and PHE (61 ± 18) and between LEU and PHE were not significant (Fig. 2B).

#### GLP‐1

None of the planned contrasts in GLP‐1 delta iAUCs during TRP (322 ± 114 pmol/L × min), LEU (−147 ± 194), PHE (−139 ± 204) or GLN (52 ± 370) infusion was significant (Fig. 2C).

#### PYY

TRP (2100 ± 672 pg/mL × min) increased plasma PYY delta iAUC more than LEU (−621 ± 445; *P* < 0.01) or GLN (75 ± 1175; *P* < 0.05), but not significantly more than PHE (1209 ± 50). PHE increased plasma PYY delta iAUC more than LEU (*P* < 0.05). There was no significant difference between LEU and GLN (Fig. 2D).

### Blood glucose, plasma insulin, and plasma glucagon

#### Blood glucose

**Figure 3:**
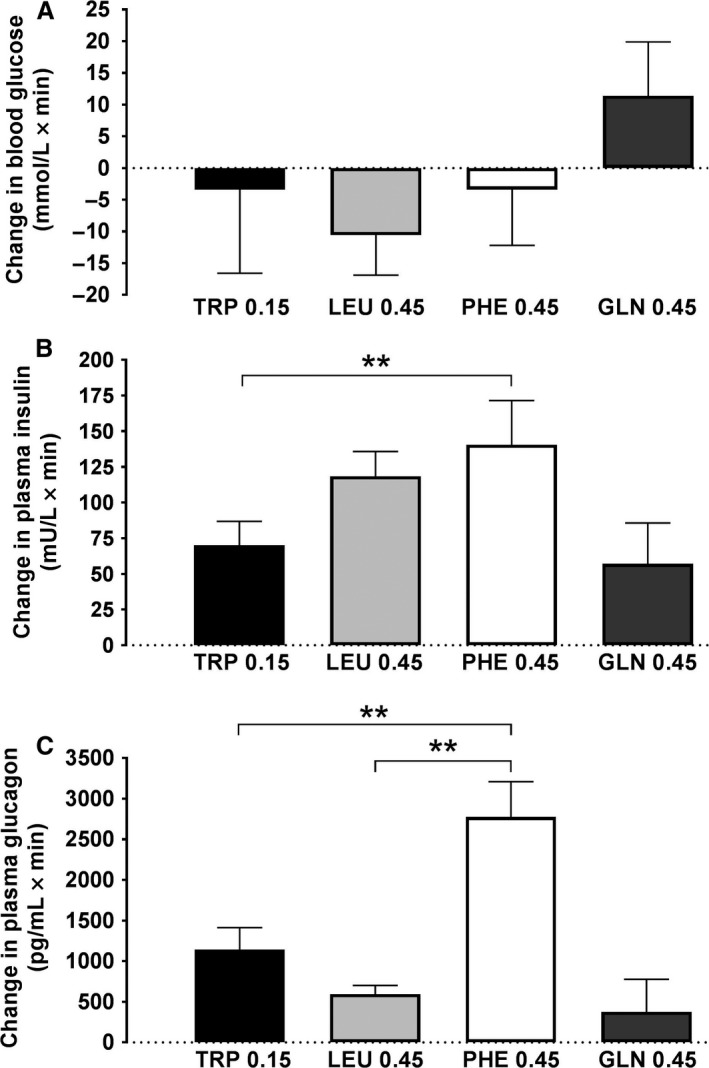
Changes in plasma insulin (A), glucagon (B) and blood glucose (C) during 90‐min intraduodenal infusions of l‐tryptophan (TRP) at 0.15 kcal/min, or l‐leucine (LEU), l‐phenylalanine (PHE) or l‐glutamine (GLN) at 0.45 kcal/min. Data are changes from respective control infusions, expressed as 90‐min incremental areas under the curve (delta iAUCs), means ± SEM; *n* = 10 for TRP and PHE, *n* = 9 for GLN, and *n* = 11 for LEU. Data were analyzed using a planned comparison approach. Two‐way analyses of variance, with delta iAUCs as the first factor and AA type as the second factor, were performed to generate experiment‐wide residual errors, which were used to compute standard errors of the difference (SED) and t‐tests, whose significances were determined using the Bonferroni–Hochberg method (Hochberg 1988), with minimum experiment‐wide two‐tailed alpha levels of 0.05. Five comparisons were performed to test whether GLN and PHE inhibit eating similarly to TRP and LEU and whether TRP inhibits eating similarly to LEU: (1) TRP versus LEU, (2) TRP versus GLN, (3) TRP versus PHE, (4) LEU versus GLN, and (5) LEU versus PHE. *P < 0.05, **P < 0.01

None of the planned contrasts in blood glucose delta iAUC during TRP (−3.4 ± 13.2 mmol/L × min), LEU (−10.6 ± 6.3), PHE (−3.4 ± 8.8) or GLN (11.4 ± 8.5) infusion was significant (Fig. 3A).

#### Insulin

PHE (140 ± 31 mU/L × min) increased plasma insulin delta iAUC more than TRP (70 ± 17; *P* < 0.05). None of the other planned contrasts was significant (LEU and GLN delta iAUCs were 118 ± 17 and 57 ± 29 mU/L × min, respectively) (Fig. 3B).

#### Glucagon

PHE (2775 ± 434 pg/mL × min) increased plasma glucagon delta iAUC more than TRP (1144 ± 270, *P* < 0.01) or LEU (594 ± 108; *P* < 0.01). None of the other planned contrasts was significant (GLN was 378 ± 399 pg/mL × min) (Fig. 3C).

## Discussion

This retrospective study evaluated the relative effects of ID infusions of four individual AAs on eating, subjective appetite and GI hormone responses in normal‐weight males. Given the increasing interest in the use of protein supplements and high‐protein diets in the management of obesity and type 2 diabetes (Larsen et al. 2010), and the phenomenon that physiological responses to proteins appear to be affected by their AA compositions (Hall et al. 2003; Anderson et al. 2004; Diepvens et al. 2008), this is an important question. Our analysis verified that, at least under the conditions tested, these AAs have significantly different effects on meal size in kcal, that is, behavioral satiation (Steinert et al. 2017). TRP, despite being given at a lower dose, reduced meal size significantly more than PHE or GLN, and LEU reduced meal size more than PHE. In addition, we found several dissociations between the patterns of significance of the AAs’ effect on eating and their effects on GI hormonal responses that shed light on mechanisms possibly underlying the differential eating‐inhibitory effects.

The greater eating‐inhibitory potency of TRP compared with PHE or GLN is especially interesting, because the TRP dose (0.15 kcal/min) was only one third of that of PHE and GLN (0.45 kcal/min). Importantly, although TRP reduced energy intake more than PHE, PHE increased glucagon and insulin delta iAUCs more than TRP. Thus, the greater eating‐inhibitory effect of TRP than PHE was not related to premeal increases of glucagon or insulin. With regard to the GI‐hormonal effects, we previously reported that this dose of ID TRP significantly increased plasma concentrations of CCK, GLP‐1 and PYY (Steinert et al. 2014). In the current analysis, TRP reduced ghrelin delta iAUCs, and increased CCK delta iAUC, more than GLN, and increased PYY delta iAUC more than either GLN or LEU. Because CCK is a well‐established satiation signal (and PYY may be as well), and ghrelin is regarded as a hunger signal (Steinert et al. 2017), it is possible that differential stimulation of these hormones may contribute to the different meal size effects, although any causal links remain to be established. We previously also found an inverse correlation between meal size and plasma TRP levels during ID TRP infusion (Steinert et al. 2014), suggesting a postabsorptive aminostatic contribution to the eating‐inhibitory effect of TRP (Frankland et al. 1956; Mellinkoff et al. 1956), possibly related to increased synthesis of 5‐hydroxy‐tryptamine (5‐HT, serotonin), which is well known for its role in the control of eating (Blundell 1977; Halford et al. 2005). Finally, it is important to recognize that gut hormones stimulated by TRP, including CCK and PYY, potently slow gastric emptying (Steinert et al. 2017), and 5‐HT is a key controller of GI motor functions (Read and Gwee 1994), thus, gastric emptying, although not measured in our study, may contribute to the eating‐inhibitory effect of TRP.

The superior satiating potency of LEU compared with PHE was associated with significantly smaller PYY and glucagon delta iAUCs. This clear dissociation indicates that average premeal increases in these hormones were not involved in the satiation effect of LEU. In addition, the superior satiating potency of LEU compared with PHE occurred in the absence of any significant differences in ghrelin, CCK, GLP‐1 or insulin delta iAUCs. Thus, these data do not support roles for these hormones in the differential satiating effects of LEU and PHE. It is important to note, however, first, that the current analysis was not sufficiently powered to permit statistical comparison of temporal changes in hormone levels (shown in Figs. [Link], [Link], [Link], [Link], [Link], [Link], [Link]), and, second, that GI‐hormonal satiation signaling may involve paracrine or other local signaling mechanisms (Steinert et al. 2017), not reflected in delta iAUCs. These possibilities warrant further evaluation. Finally, postabsorptive mechanisms may mediate LEU‐induced satiation. In support, we previously found that plasma LEU concentrations increased substantially relative to control following ID LEU infusion (Steinert et al. 2015a), and both hypothalamic (Cota et al. 2006; Morrison et al. 2007) and brainstem (Blouet and Schwartz 2012) LEU sensitivity have been linked to the inhibition of eating.

That PHE inhibited eating less than TRP, despite similar increases in plasma CCK, GLP‐1, PYY and glucagon, is surprising and does not support the general importance of these hormones in the relative effects of these AAs on eating. As mentioned above, it is important to note, however, that the current analysis was not sufficiently powered to permit statistical comparison of momentary hormone levels (see Figs. [Link], [Link], [Link], [Link], [Link], [Link], [Link]), which may contribute to the AAs’ differential effects on appetite and eating. Additional dissociations involving PHE were that PHE increased plasma glucagon more than TRP or LEU and increased plasma insulin more than TRP, although both TRP and LEU reduced energy intake more than PHE. This is surprising given the evidence that both glucagon and insulin can contribute to the inhibition of eating in humans or nonhuman primates in other test situations (Woods et al. 1979; Geary et al. 1992). Again, however, tests of glucagon and insulin levels at potentially critical moments, such as at meal onset or meal end, would be required to more fully investigate their potential contributions.

Interestingly, the differences in the eating‐inhibitory efficacies of AAs were not closely associated with the appetite delta iAUCs. For example, LEU reduced meal size markedly more, but reduced premeal prospective consumption significantly less, than PHE. LEU also reduced prospective food consumption less than TRP, but these two AAs did not differ in their effects on eating. Our analyses revealed no differences in the effects of AAs on other appetite ratings, possibly because our study was not powered to detect between‐subjects differences (Flint et al. 2000). It is also important to note that, although appetite VAS are sensitive to experimental manipulations and reproducible (Flint et al. 2000; Parker et al. 2004; Stubbs et al. 2007), they have failed to predict meal size under some conditions (Flint et al. 2000; Stubbs et al. 2007).

The effects of AAs on glycemic control are complex and were not a primary focus of our studies. We previously found a small blood glucose‐lowering, insulinotropic effect of LEU and a small insulinotropic effect of TRP (Steinert et al. 2014, 2015a), but did not find any differences in effects between AAs in the current study. However, we only investigated the differential effects of AAs on “fasting” blood glucose. Determination of the effects of AAs on meal‐related glycemia, that is, using an experimental design with concomitant administration of carbohydrates, may have resulted in a different outcome, and warrants further evaluation.

Our study has a number of limitations that require consideration. (1) As outlined, this study was an analysis of data from four separate studies, rather than one prospective cross‐over study encompassing all four AAs. Although identically designed, TRP was given at a lower dose (yet was substantially more satiating). (2) To what extent our findings apply to more physiological conditions under which foods high in specific AAs are ingested is not known. (3) We ended infusions before the test meals; infusions continued into the meal may have revealed additional satiation effects. (4) We offered test meals at a specific time, rather than when the subjects were ready to eat; the latter design may have revealed effects on postprandial satiety. (5) Our study was also not powered to analyze absolute hormone levels at each measurement time. We observed prompt secretion of CCK in response to TRP and LEU within 30 min upon start of the infusions (Steinert et al. 2014, 2015a), while GLP‐1 secretion with TRP was delayed, with elevated concentrations occurring between 75–90 min (Steinert et al. 2014). Such differences may differentially affect food intake (Steinert et al. 2017). (6) We did not analyze temporal dynamics of changes in plasma AA concentrations, such as relative rates of change, which would provide additional information on aminostatic controls of eating.

In conclusion, ID infusions of TRP (at 0.15 kcal/min), LEU, PHE and GLN (all three at 0.45 kcal/min) produce differential effects on food intake and GI and pancreatic endocrine responses. The greater satiating efficacy of LEU versus PHE was significantly dissociated from their effects on PYY, while the greater satiating potency of TRP versus PHE was significantly dissociated from their effects on insulin and glucagon. In contrast, ghrelin and CCK, and potentially other mechanisms, including neural sensing of individual AAs, appear to be stronger candidate mechanisms for the relative satiating effects obtained. This warrants evaluation in prospective studies.

## Conflict of Interest

All authors disclose that they do not have any financial or personal relationships with other people or organizations that could inappropriately influence (bias) this work.

## Data Accessibility

## Supporting information




**Figure S1.** Diagram of participant flow through the studies.

**Figure S2.** Plasma ghrelin concentrations during 90‐min ID infusions of l‐tryptophan (TRP) at 0.15 kcal/min, or l‐leucine (LEU), l‐phenylalanine (PHE) or l‐glutamine (GLN) at 0.45 kcal/min or respective controls (A–D).

**Figure S3.** Plasma cholecystokinin (CCK) concentrations during 90‐min ID infusions of l‐tryptophan (TRP) at 0.15 kcal/min, or l‐leucine (LEU), l‐phenylalanine (PHE) or l‐glutamine (GLN) at 0.45 kcal/min or respective controls (A–D). For statistical comparisons, incremental areas under the curve (iAUCs) were calculated for each AA and its control infusion under the profiles (*t* = 0 to 90 min) and data expressed as changes in iAUC for each AA relative to control infusion (see Fig. 2).

**Figure S4.** Plasma glucagon‐like peptide‐1 (GLP‐1) concentrations during 90‐min ID infusions of l‐tryptophan (TRP) at 0.15 kcal/min, or l‐leucine (LEU), l‐phenylalanine (PHE) or l‐glutamine (GLN) at 0.45 kcal/min or respective controls (A–D).

**Figure S5.** Peptide YY (PYY) concentrations during 90‐min ID infusions of l‐tryptophan (TRP) at 0.15 kcal/min, or l‐leucine (LEU), l‐phenylalanine (PHE) or l‐glutamine (GLN) at 0.45 kcal/min or respective controls (A–D).

**Figure S6.** Blood glucose concentrations during 90‐min ID infusions of l‐tryptophan (TRP) at 0.15 kcal/min, or l‐leucine (LEU), l‐phenylalanine (PHE) or l‐glutamine (GLN) at 0.45 kcal/min or respective controls (A–D).

**Figure S7.** Plasma insulin concentrations during 90‐min ID infusions of l‐tryptophan (TRP) at 0.15 kcal/min, or l‐leucine (LEU), l‐phenylalanine (PHE) or l‐glutamine (GLN) at 0.45 kcal/min or respective controls (A‐D).

**Figure S8.** Plasma glucagon concentrations during 90‐min ID infusions of l‐tryptophan (TRP) at 0.15 kcal/min, or l‐leucine (LEU), l‐phenylalanine (PHE) or l‐glutamine (GLN) at 0.45 kcal/min or respective controls (A–D).
