# Supplementary material for: Comparative effects of intraduodenal amino acid infusions on food intake and gut hormone release in healthy males
Source: Physiol Rep. 2017 Nov 15;5(21):e13492. doi: 10.14814/phy2.13492 (PMC5688783; doi:10.14814/phy2.13492)
Supplement: Supplementary file 1 — Figure S1. Diagram of participant flow through the studies. [file PHY2-5-e13492-s001.doc]

**CONSORT 2010 Flow Diagram**

**Allocation**

**Analysis**

**Enrollment**

Assessed for eligibility (n=44)

Randomized (n=44)

TRP study: n=12
LEU study: n=12
PHE study: n=10
GLN study: n=10

Analyzed (n=40)

Allocated to intervention (n=44)

♦ Received allocated intervention (n=44)

Excluded (n=2, both from TRP study)

♦  withdrew due to side effects including nausea/vomiting (n=1) or dizziness (n=1)

Excluded (n=2) (outliers/missing data)

♦  n=1 (LEU study)

♦ n=1 (GLN study)

TRP study: n=12
LEU study: n=12
PHE study: n=10
GLN study: n=10

TRP study: n=10
LEU study: n=11
PHE study: n=10
GLN study: n=9
