# Supplementary figures and images for: Comparative effects of intraduodenal amino acid infusions on food intake and gut hormone release in healthy males
Source: Physiol Rep. 2017 Nov 15;5(21):e13492. doi: 10.14814/phy2.13492 (PMC5688783; doi:10.14814/phy2.13492)

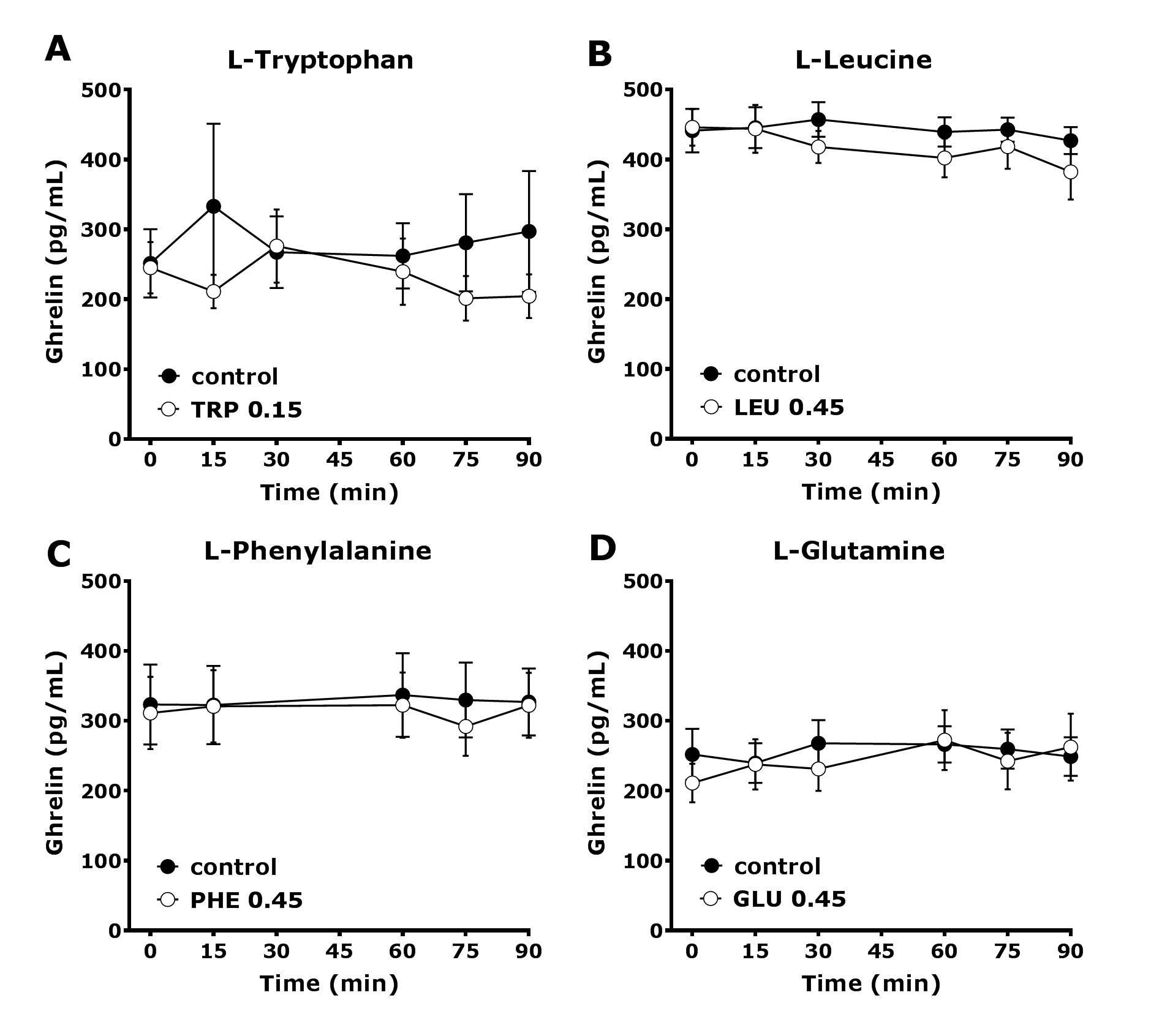

Supplement: Supplementary file 2 — Figure S2. Plasma ghrelin concentrations during 90‐min ID infusions of l‐tryptophan (TRP) at 0.15 kcal/min, or l‐leucine (LEU), l‐phenylalanine (PHE) or l‐glutamine (GLN) at 0.45 kcal/min or respective controls (A–D). [file PHY2-5-e13492-s002.tif]

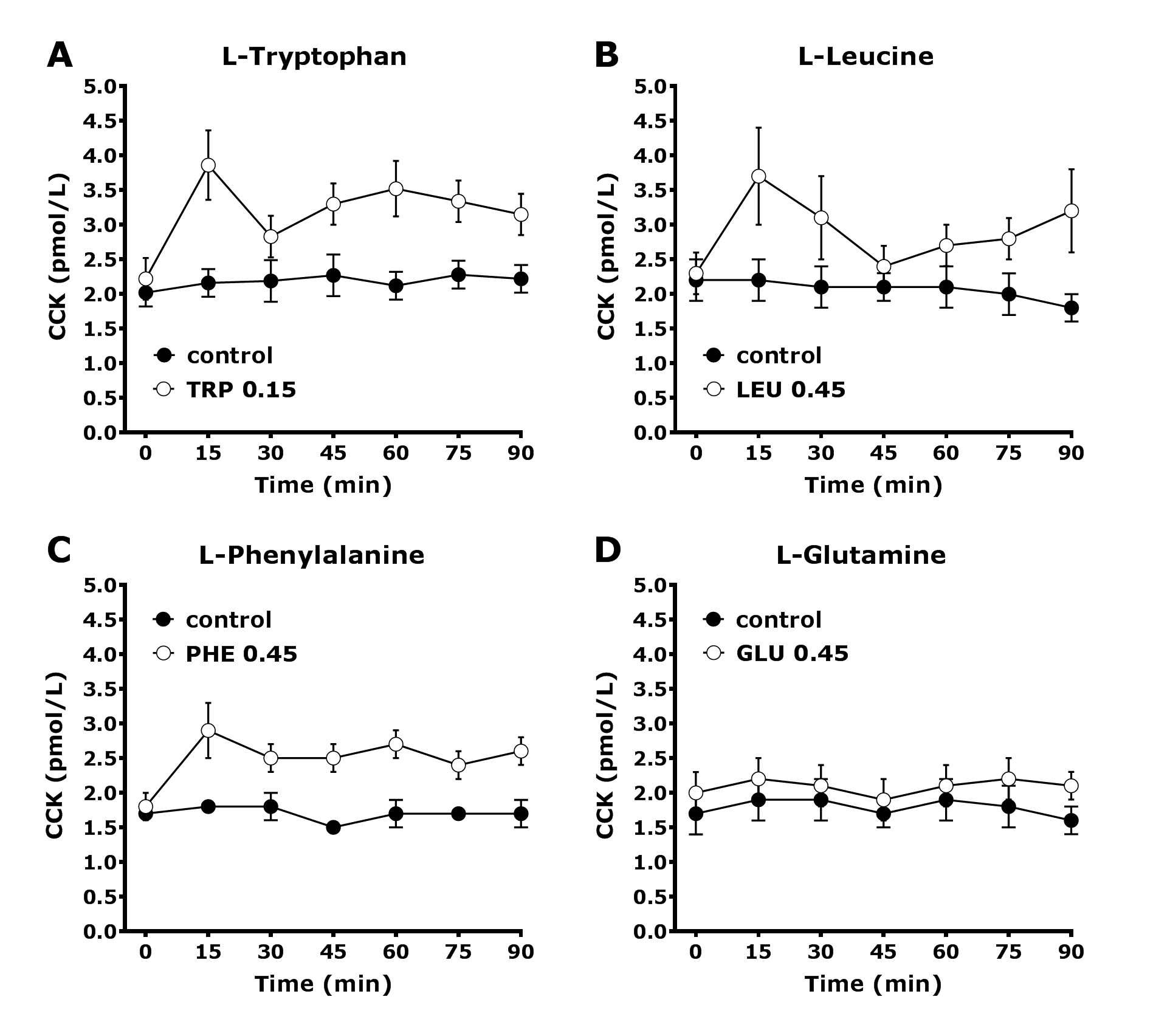

Supplement: Supplementary file 3 — Figure S3. Plasma cholecystokinin (CCK) concentrations during 90‐min ID infusions of l‐tryptophan (TRP) at 0.15 kcal/min, or l‐leucine (LEU), l‐phenylalanine (PHE) or l‐glutamine (GLN) at 0.45 kcal/min or respective controls (A–D). For statistical comparisons, incremental areas under the curve (iAUCs) were calculated for each AA and its control infusion under the profiles (t = 0 to 90 min) and data expressed as changes in iAUC for each AA relative to control infusion (see Fig. 2). [file PHY2-5-e13492-s003.tif]

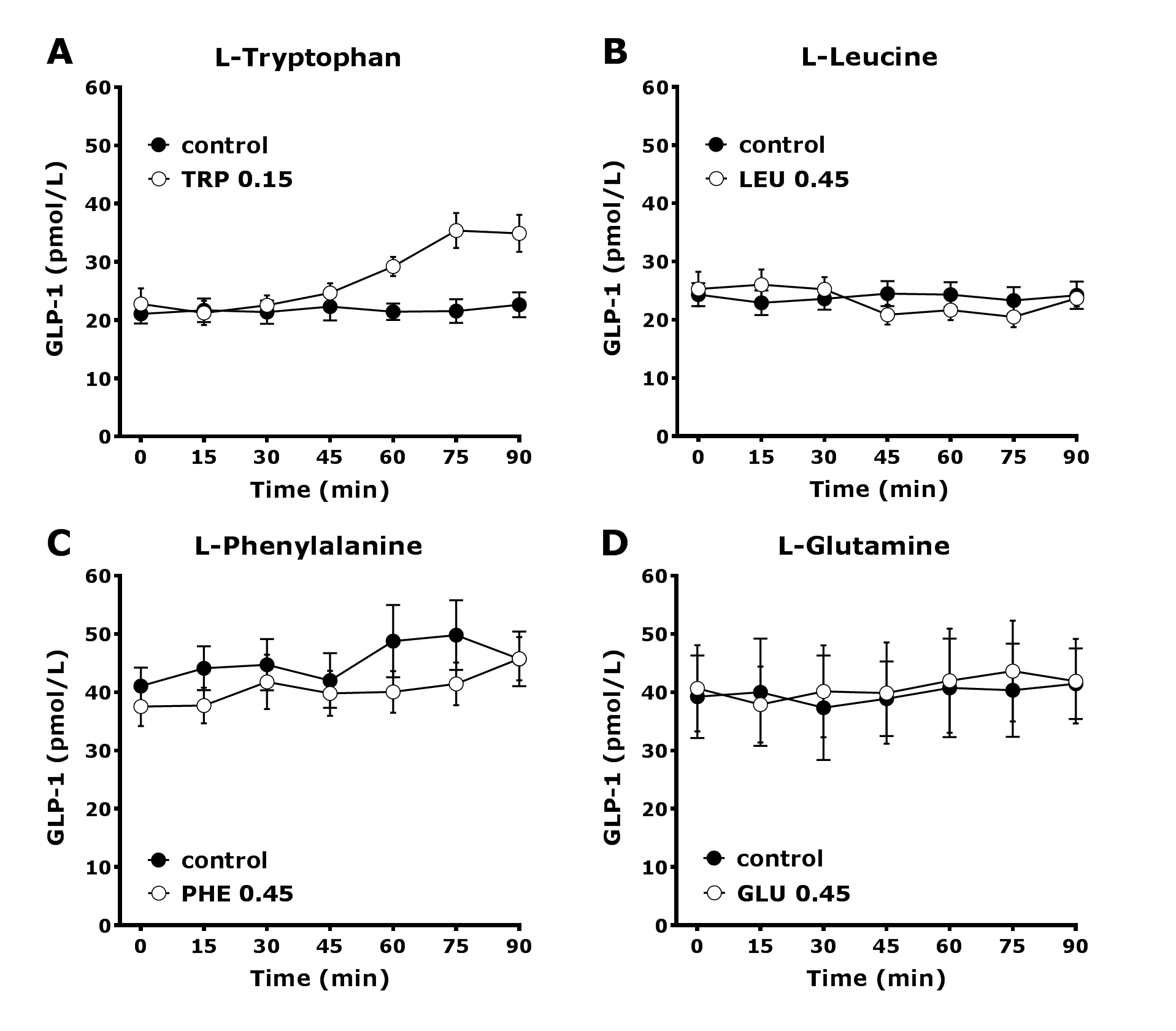

Supplement: Supplementary file 4 — Figure S4. Plasma glucagon‐like peptide‐1 (GLP‐1) concentrations during 90‐min ID infusions of l‐tryptophan (TRP) at 0.15 kcal/min, or l‐leucine (LEU), l‐phenylalanine (PHE) or l‐glutamine (GLN) at 0.45 kcal/min or respective controls (A–D). [file PHY2-5-e13492-s004.tif]

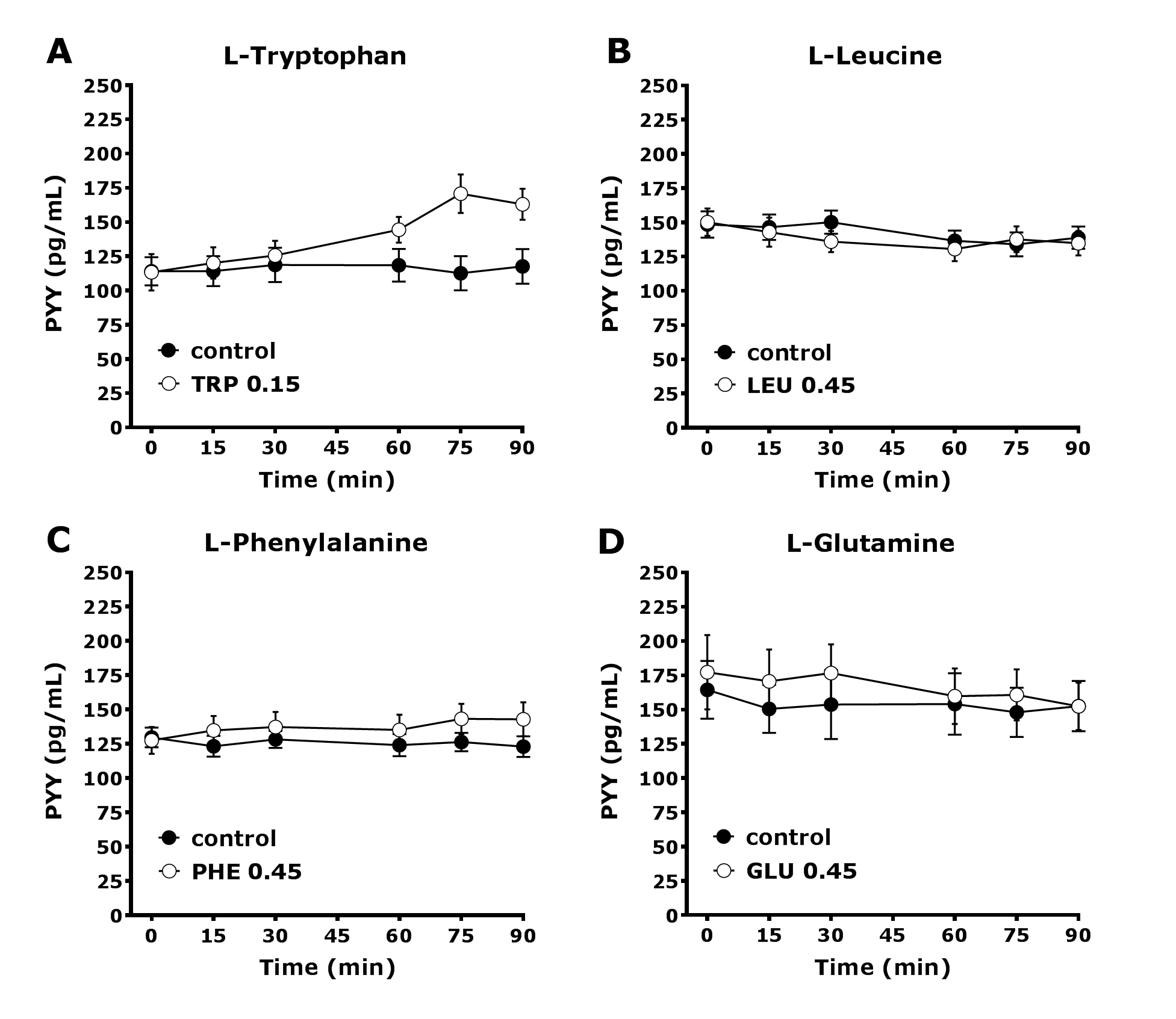

Supplement: Supplementary file 5 — Figure S5. Peptide YY (PYY) concentrations during 90‐min ID infusions of l‐tryptophan (TRP) at 0.15 kcal/min, or l‐leucine (LEU), l‐phenylalanine (PHE) or l‐glutamine (GLN) at 0.45 kcal/min or respective controls (A–D). [file PHY2-5-e13492-s005.tif]

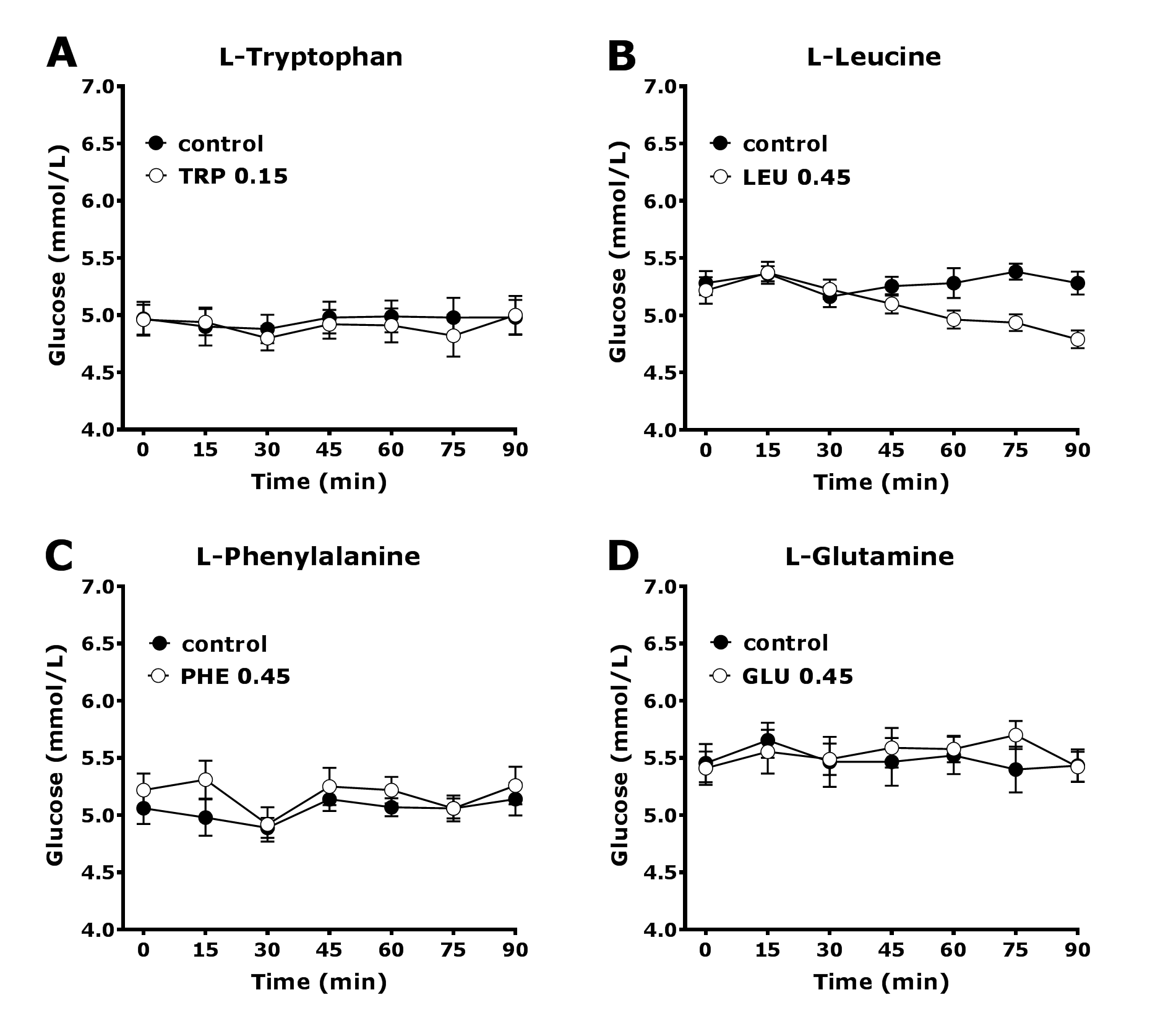

Supplement: Supplementary file 6 — Figure S6. Blood glucose concentrations during 90‐min ID infusions of l‐tryptophan (TRP) at 0.15 kcal/min, or l‐leucine (LEU), l‐phenylalanine (PHE) or l‐glutamine (GLN) at 0.45 kcal/min or respective controls (A–D). [file PHY2-5-e13492-s006.tif]

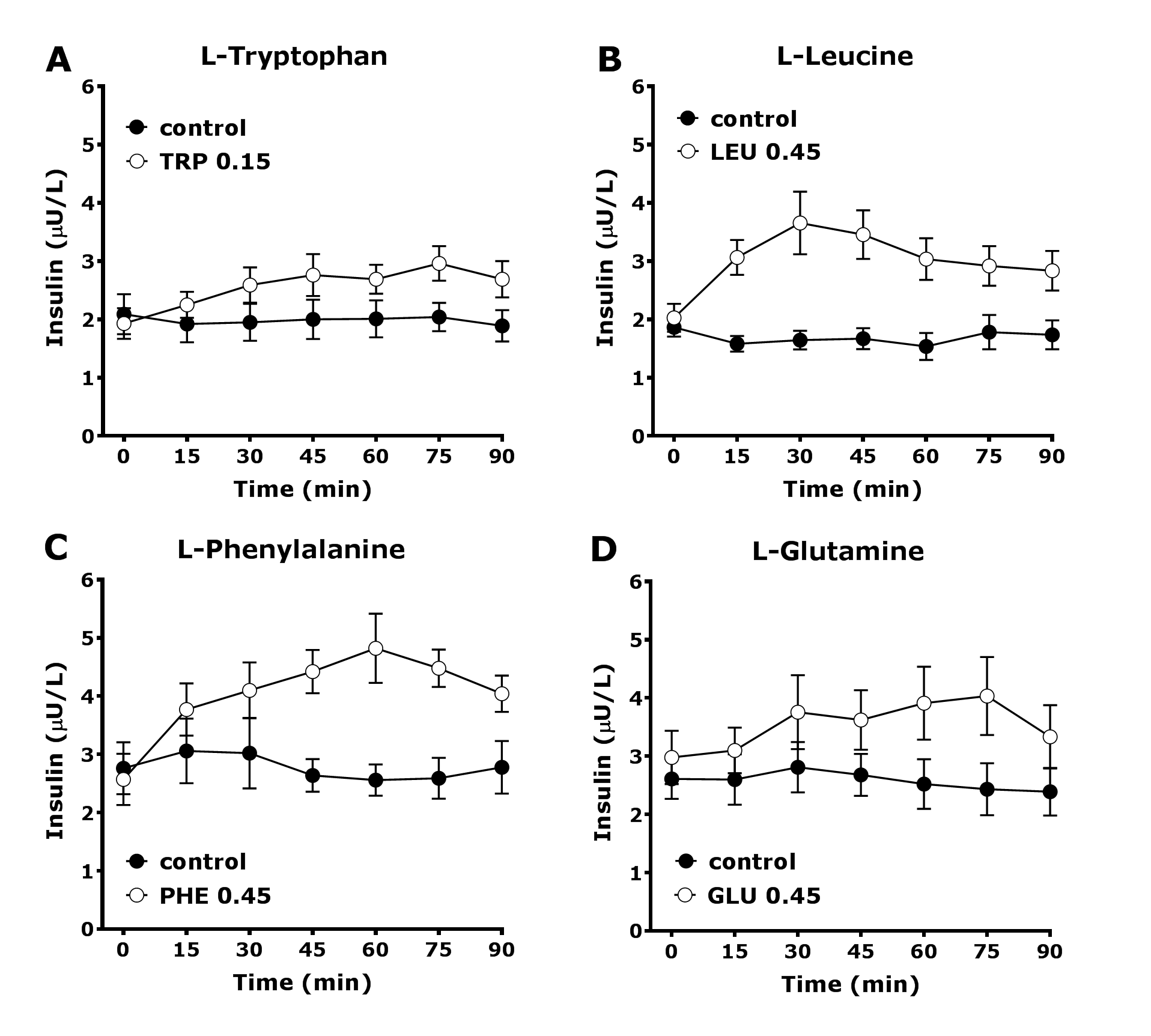

Supplement: Supplementary file 7 — Figure S7. Plasma insulin concentrations during 90‐min ID infusions of l‐tryptophan (TRP) at 0.15 kcal/min, or l‐leucine (LEU), l‐phenylalanine (PHE) or l‐glutamine (GLN) at 0.45 kcal/min or respective controls (A‐D). [file PHY2-5-e13492-s007.tif]

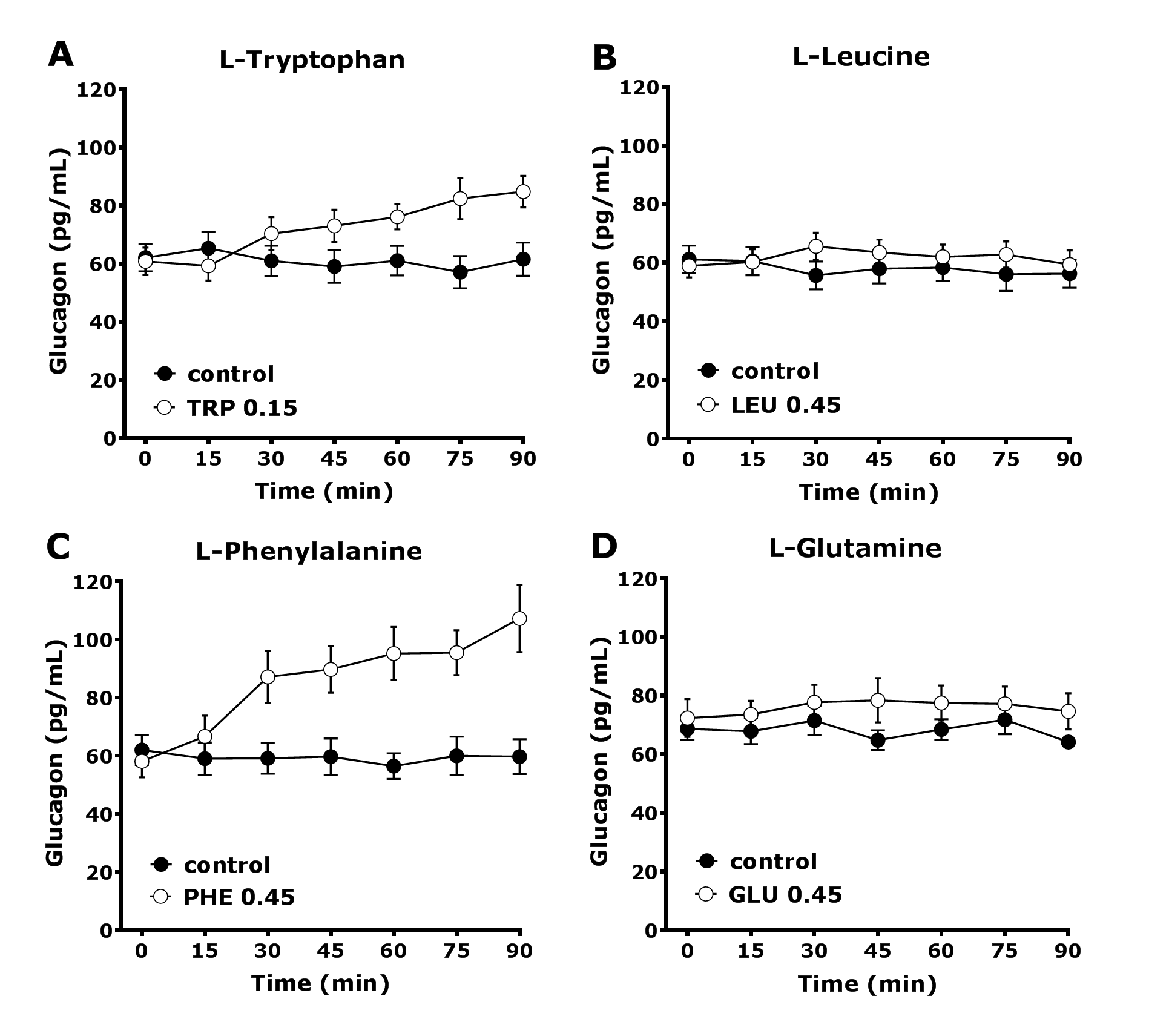

Supplement: Supplementary file 8 — Figure S8. Plasma glucagon concentrations during 90‐min ID infusions of l‐tryptophan (TRP) at 0.15 kcal/min, or l‐leucine (LEU), l‐phenylalanine (PHE) or l‐glutamine (GLN) at 0.45 kcal/min or respective controls (A–D). [file PHY2-5-e13492-s008.tif]
